# Supplementary material for: Direct RNA sequencing coupled with adaptive sampling enriches RNAs of interest in the transcriptome
Source: Nat Commun. 2024 Jan 11;15:481. doi: 10.1038/s41467-023-44656-3 (PMC10784512; doi:10.1038/s41467-023-44656-3)
Supplement: Supplementary file 1 — Supplementary Information [file 41467_2023_44656_MOESM1_ESM.docx]

**Supplementary Information**

**Direct RNA sequencing coupled with adaptive sampling enriches RNAs of interest in the transcriptome**


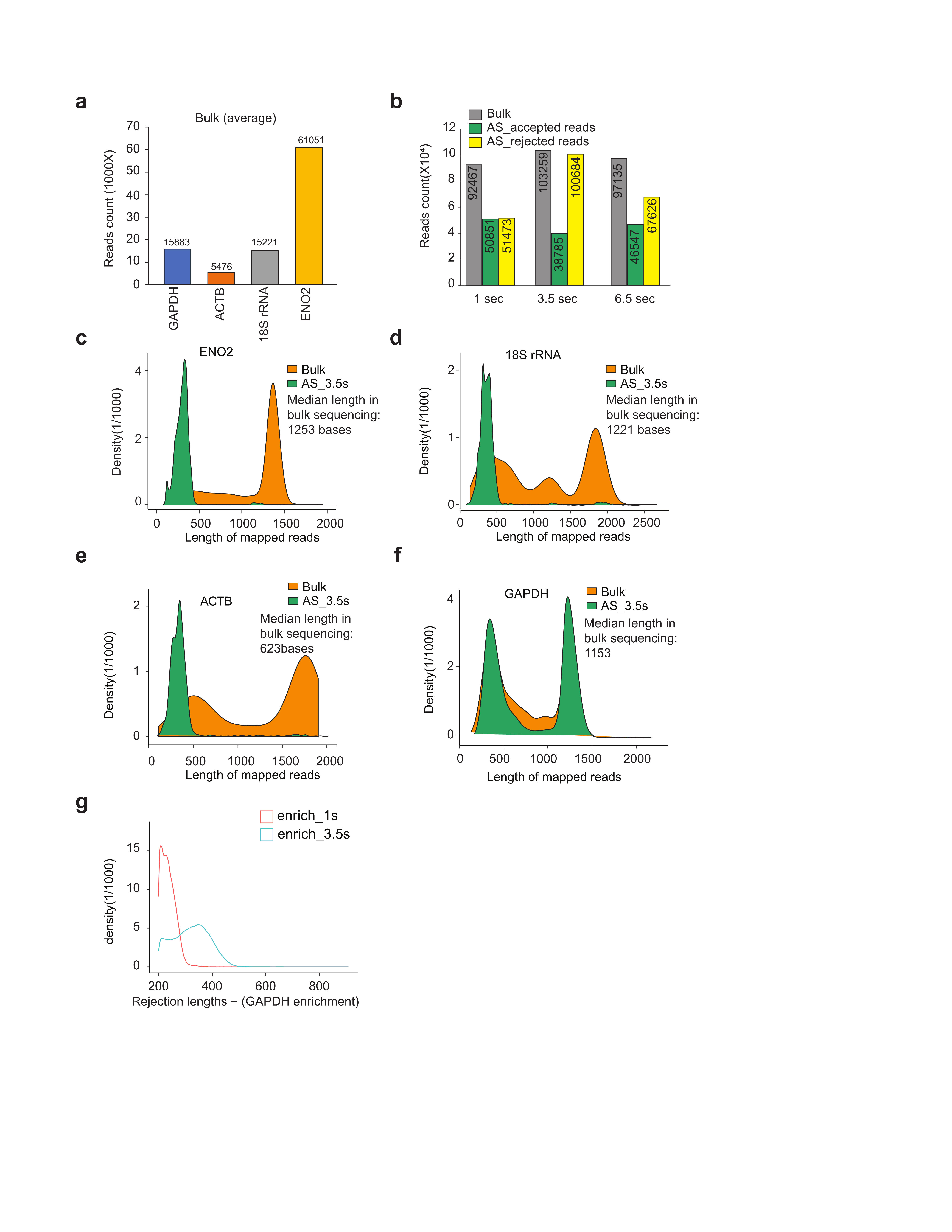


**Supplementary Figure 1. Direct RNA sequencing of the IVT pool. a**, Bar plots showing the number of reads of GAPDH, ENO2, 18S rRNA, and ACTB present in bulk sequencing. **b**, Bar plots showing the reads count from bulk and 1sec, 3.5sec and 6.5sec adaptive sequencing. **c-f**, Density plots showing the length of mapped reads of ENO2 (**c**), 18S rRNA (**d**), ACTB (**e**), and GAPDH (**f**) in bulk sequencing and during adaptive sequencing. **g**, Density plots showing the distribution of read lengths in the rejected read pool when 1 or 3.5 sec decision time is used in adaptive sampling. The color labels are as shown.


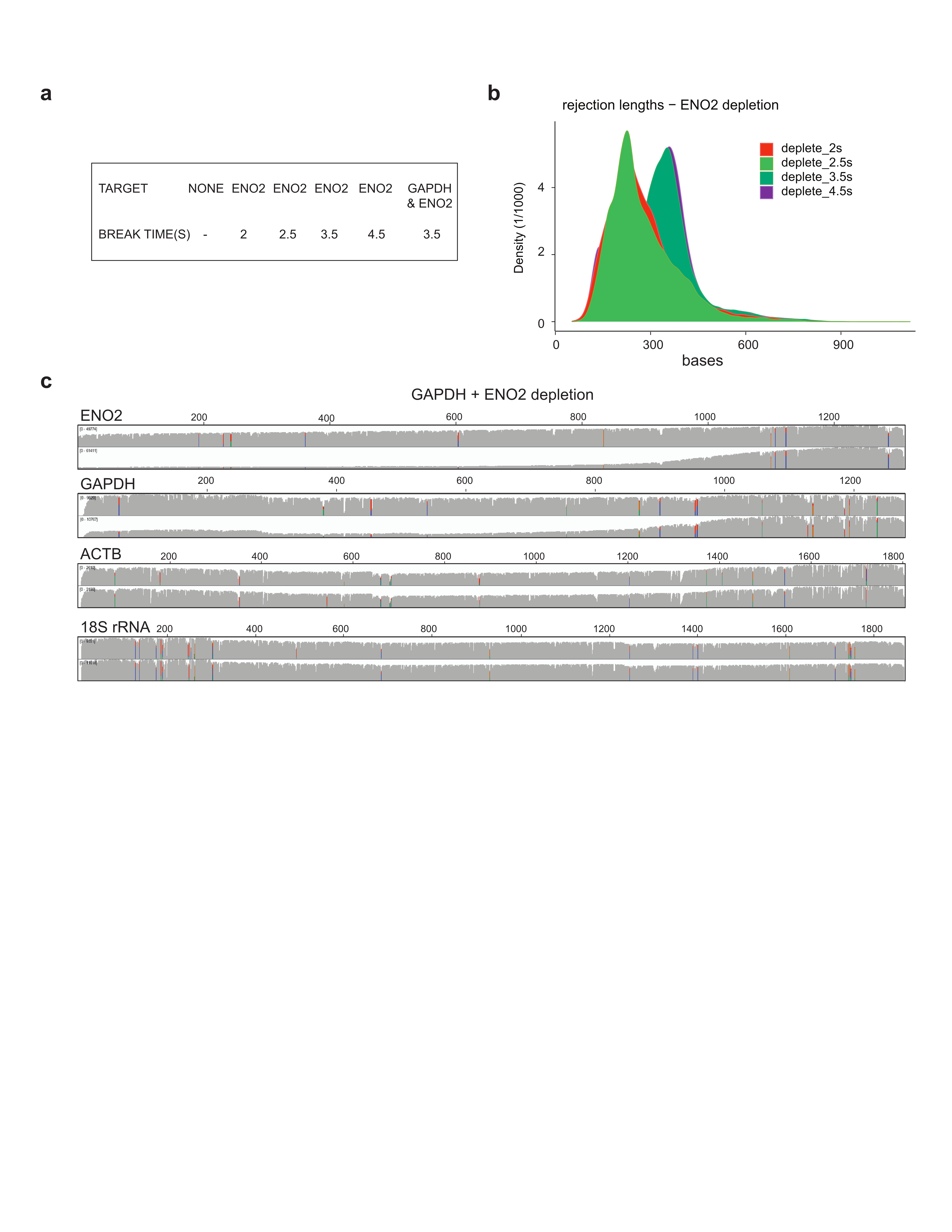


**Supplementary Figure 2. Using adaptive sampling to deplete transcripts of interest. a**, Table showing the parameters for break times (decision times) tested in the depletion mode of adaptive sampling. **b**, Density plots showing the distribution of read lengths of rejected reads under different decision times of adaptive sampling. The color labels are as shown. **c**, IGV plots showing the distribution of read coverage along the length of GAPDH, ACTB, 18S rRNA, and ENO2 after GAPDH and ENO2 depletion by adaptive sampling.


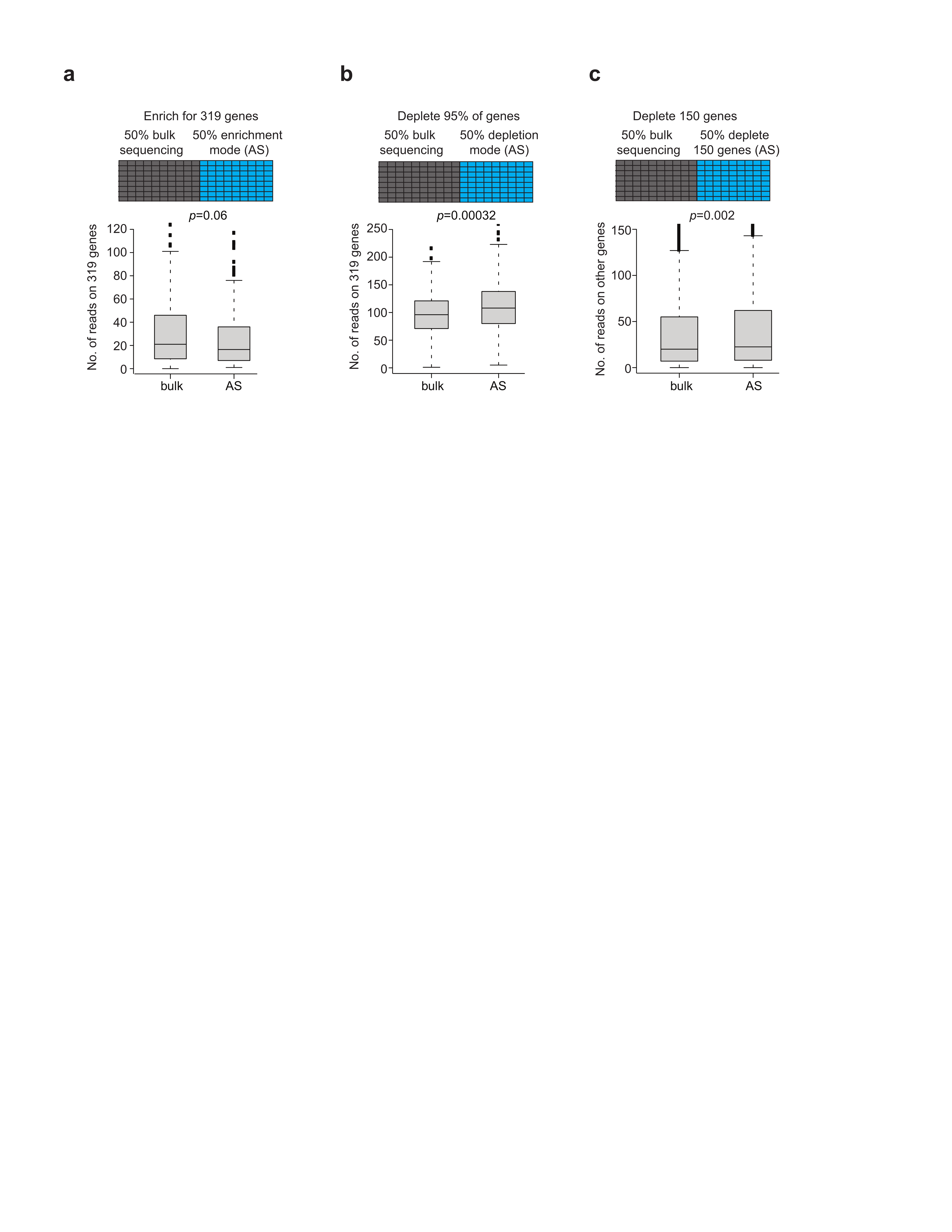


**Supplementary Figure 3. Applying adaptive sampling to enrich transcripts in the *Candida albicans* transcriptome. a,** Top: schematic showing that 50% of the pores of a flow cell are used for bulk sequencing and 50% of the pores are used for adaptive sampling. Bottom: Boxplot showing the number of reads that mapped to each of the 319 transcripts in bulk sequencing versus in the enrichment mode of adaptive sampling. *P*-value is calculated using Wilcoxon Rank Sum Test. **b**, Top: schematic showing that 50% of the pores of a flow cell are used for bulk sequencing and 50% of the pores are used for adaptive sampling to deplete 95% of the transcripts. Bottom: Boxplot showing the number of reads that mapped to the 319 transcripts in bulk sequencing versus in the depletion mode of adaptive sampling. **c**, Top: schematic showing that 50% of the pores of a flow cell are used for bulk sequencing and 50% of the pores are used for adaptive sampling to deplete the top 150 transcripts. Bottom: Boxplot showing the number of reads that mapped to other transcripts in bulk sequencing versus in the depletion mode of adaptive sampling after depleting the top 150 transcripts. *P*-value is calculated using Wilcoxon Rank Sum Test.


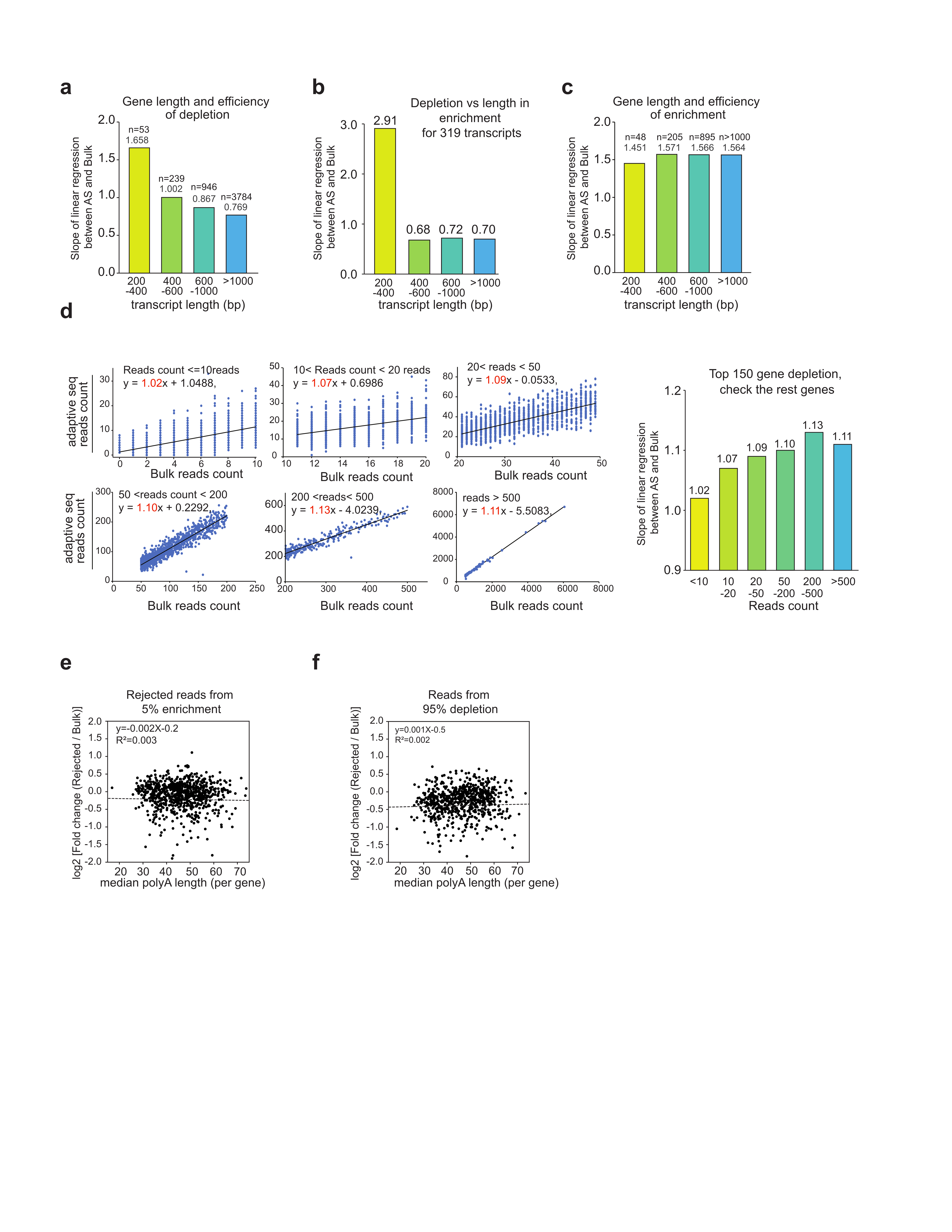


**Supplementary Figure 4. Features of RNA that enable enrichment of RNAs in the transcriptome. a**, Bar plots showing the slope of linear regression of reads count belonging to the depleted 4997 transcripts of different transcript lengths (200-400bp, 400-600bp, 600-1000bp, >1000bp) in in adaptive sampling and bulk sequencing. **b**, Bar plots showing the slope of linear regression of reads count for the remaining transcripts after 319 transcripts are enriched in adaptive sampling and Bulk. The remaining transcripts are binned according to their different transcript lengths (200-400bp, 400-600bp, 600-1000bp, >1000bp) in bulk sequencing. **c**, Bar plots showing the slope of linear regression of reads count belonging to the enriched 319 transcripts in adaptive sampling versus bulk sequencing, the 319 genes are binned according to their transcript lengths (200-400bp, 400-600bp, 600-1000bp, >1000bp) in bulk sequencing. **d**, Scatterplots showing the reads belonging to non-depleted genes of different abundance (<10, 10-20, 20-50, 50-200, >500 reads) in Adaptive sampling(*Y*-axis) and Bulk (*X*-axis), after depletion of top 150 abundant genes (*Left*). Bar plots showing the slope of linear regression of reads count between adaptive sampling and Bulk belonging to the rest of the transcriptome, after the top 150 transcripts are depleted, binned by transcript abundance (*Right*). **e, f**, Scatterplots showing the correlation between the efficiency of depletion and polyA tail length, either enriching for 5% of transcripts (e) or depleting 95% of transcripts (f).


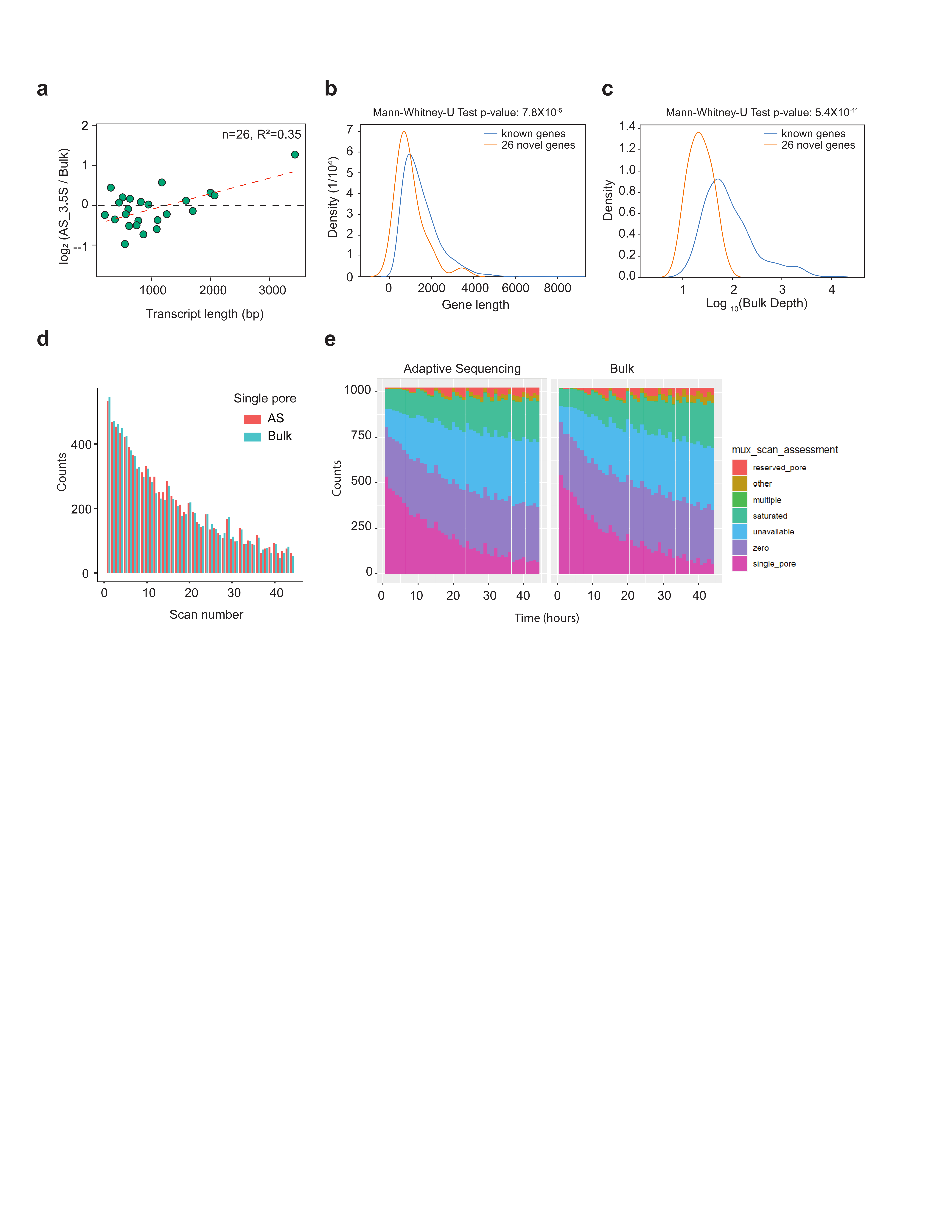


**Supplementary Figure 5. Adaptive sampling does not impact pore health.**  **a**, Scatterplot showing the log2 ratio of reads in adaptive sampling over bulk sequencing for the 26 newly identified transcripts versus the length of the transcripts. **b**, Density plot showing the distribution of the length of the newly identified transcripts versus the length of other annotated transcripts in *Candida albicans*. **c**, Density plot showing the distribution of the abundance of the novel transcripts versus the abundance of other annotated transcripts in *Candida albicans*. p-values were calculated by Mann-Whitney-U Test. **d**, Bar plots showing the number of available pores over scan frequencies, single pores refer to pores that are available for sequencing. **e**, Detailed status of pores over the lifetime of the sequencing run. The *X*-axis corresponds to mux scan number (scan frequency is 1.5 hours) and the *Y*-axis indicates the number of pores.
