# Supplementary material for: Direct RNA sequencing coupled with adaptive sampling enriches RNAs of interest in the transcriptome
Source: Nat Commun. 2024 Jan 11;15:481. doi: 10.1038/s41467-023-44656-3 (PMC10784512; doi:10.1038/s41467-023-44656-3)
Supplement: Supplementary file 4 — Reporting Summary [file 41467_2023_44656_MOESM4_ESM.pdf]

nature portfolio

Corresponding author(s): Wan Yue

Last updated by author(s): Dec 11, 2023

Reporting Summary

Nature Portfolio wishes to improve the reproducibility of the work that we publish. This form provides structure for consistency and transparency in reporting. For further information on Nature Portfolio policies, see our Editorial Policies and the Editorial Policy Checklist.

Statistics

For all statistical analyses, confirm that the following items are present in the figure legend, table legend, main text, or Methods section.

n/a	Confirmed	
☐	☒	The exact sample size (*n*) for each experimental group/condition, given as a discrete number and unit of measurement
☐	☒	A statement on whether measurements were taken from distinct samples or whether the same sample was measured repeatedly
☐	☒	The statistical test(s) used AND whether they are one- or two-sided<br>*Only common tests should be described solely by name; describe more complex techniques in the Methods section.*
☐	☒	A description of all covariates tested
☐	☒	A description of any assumptions or corrections, such as tests of normality and adjustment for multiple comparisons
☒	☐	A full description of the statistical parameters including central tendency (e.g. means) or other basic estimates (e.g. regression coefficient) AND variation (e.g. standard deviation) or associated estimates of uncertainty (e.g. confidence intervals)
☐	☒	For null hypothesis testing, the test statistic (e.g. *F*, *t*, *r*) with confidence intervals, effect sizes, degrees of freedom and *P* value noted<br>*Give P values as exact values whenever suitable.*
☒	☐	For Bayesian analysis, information on the choice of priors and Markov chain Monte Carlo settings
☒	☐	For hierarchical and complex designs, identification of the appropriate level for tests and full reporting of outcomes
☒	☐	Estimates of effect sizes (e.g. Cohen's *d*, Pearson's *r*), indicating how they were calculated

Our web collection on statistics for biologists contains articles on many of the points above.

Software and code

Policy information about availability of computer code

Data collection	Adaptive sampling runs were set up in MinKNOW GUI (versions 22.08.9 and 22.10.5), with modifications to decision times made to "/opt/ont/minknow/conf/package/sequencing/sequencing_MIN106_RNA.toml" under the `break_time_in_seconds` parameter. For depletion runs, an additional parameter modification was applied (deplete_stop_receiving_min_sequence_length = 600). Live basecalling was performed using Guppy 6.2.11 or 6.3.8, using the high accuracy (HAC) model. Enrichment or depletion targets were provided as indexed FASTA files, using the minimap2 indexing preset for short reads. For adaptive sampling decision time testing, library prepared from IVT RNA mixes were run for about 25 minutes each, either under regular bulk sequencing or with various decision time parameters. For candida albicans experiments, each MinION flow cell was divided in half, with 256 channels running regular bulk sequencing and the other 256 channels performing adaptive sampling with the appropriate gene panels. Runs were stopped at either the 48-hour or 68-hour mark.
Data analysis	FASTQ output files that passed the Q-score threshold value of 7 were used for data analysis. Output from adaptive sampling were grouped based on adaptive sampling decisions as reported in the "adaptive_sampling_[flow cell id]_[run id].csv" file generated during the run, with `stop_receiving` or `no_decision` reads included in the accepted pool and `unblock` reads included in the rejected pool. In the split flow cell setup for candida albicans experiments, reads were first assigned to regular bulk sequencing or adaptive sampling channels utilising read_id and channel information provided in the "sequencing_summary_[flow cell id]_[run id].txt" file. Reads in the respective categories were aligned to a reference consisting of sequences of IVT transcripts or a reference transcriptome for candida albicans using minimap2 (version 2.24) under the option `-ax map-ont`. Aligned reads were subsequently filtered using SAMtools (version 1.14) with the -F 2308 flag to remove unmapped, supplementary and secondary alignments.

For manuscripts utilizing custom algorithms or software that are central to the research but not yet described in published literature, software must be made available to editors and reviewers. We strongly encourage code deposition in a community repository (e.g. GitHub). See the Nature Portfolio guidelines for submitting code & software for further information.

Data

Policy information about availability of data

All manuscripts must include a data availability statement. This statement should provide the following information, where applicable:

- Accession codes, unique identifiers, or web links for publicly available datasets
- A description of any restrictions on data availability
- For clinical datasets or third party data, please ensure that the statement adheres to our policy

The raw sequences data was uploaded to European Nucleotide Archive https://www.ebi.ac.uk/ena/browser/home. The accession numbers are uploaded and archived at ENA with accession PRJEB70914. All data generated in this study is available in Article and Supplementary Data.

Research involving human participants, their data, or biological material

Policy information about studies with human participants or human data. See also policy information about sex, gender (identity/presentation), and sexual orientation and race, ethnicity and racism.

Reporting on sex and gender	N/A
Reporting on race, ethnicity, or other socially relevant groupings	N/A
Population characteristics	N/A
Recruitment	N/A
Ethics oversight	N/A

Note that full information on the approval of the study protocol must also be provided in the manuscript.

Field-specific reporting

Please select the one below that is the best fit for your research. If you are not sure, read the appropriate sections before making your selection.

☒ Life sciences ☐ Behavioural & social sciences ☐ Ecological, evolutionary & environmental sciences

For a reference copy of the document with all sections, see nature.com/documents/nr-reporting-summary-flat.pdf

Life sciences study design

All studies must disclose on these points even when the disclosure is negative.

Sample size	Information on the the size of the datasets is available in the Methods section and supplemental tables. No statistical methods were employed to determine sample size.
Data exclusions	No data exclusion
Replication	We provide the code necessary for replicating the results. Different package versions or computational environments might lead to slightly different outputs.
Randomization	Ransom test were used to train different Nanopore sequencing parameters in order to evaluate them, as common practice.
Blinding	Blinding is not applicable to our work, since our main finding is the development of a computational tool.

Reporting for specific materials, systems and methods

We require information from authors about some types of materials, experimental systems and methods used in many studies. Here, indicate whether each material, system or method listed is relevant to your study. If you are not sure if a list item applies to your research, read the appropriate section before selecting a response.

Materials & experimental systems

n/a	Involved in the study
☒	☐ Antibodies
☐	☒ Eukaryotic cell lines
☒	☐ Palaeontology and archaeology
☒	☐ Animals and other organisms
☒	☐ Clinical data
☒	☐ Dual use research of concern
☒	☐ Plants

Methods

n/a	Involved in the study
☒	☐ ChIP-seq
☒	☐ Flow cytometry
☒	☐ MRI-based neuroimaging

Eukaryotic cell lines

Policy information about cell lines and Sex and Gender in Research

Cell line source(s)	candida albicans
Authentication	*Describe the authentication procedures for each cell line used OR declare that none of the cell lines used were authenticated.*
Mycoplasma contamination	Tested Negative
Commonly misidentified lines (See ICLAC register)	*Name any commonly misidentified cell lines used in the study and provide a rationale for their use.*
